# Grain arsenic accumulation is independent of agronomic traits in rice under field conditions

**DOI:** 10.1007/s12298-025-01597-z

**Published:** 2025-06-21

**Authors:** Parminder Singh Saini, Manish Pandey, Samrath Baghel, Suraj Patil, Parmeshwar Kumar Sahu, Vinay Kumar, Bikram Kishore Das, Deepak Sharma, Ashish Kumar Srivastava

**Affiliations:** 1 Department of Genetics and Plant Breeding, Indira Gandhi Krishi Vishwavidyalaya, Raipur, 492012 India; 2 Nuclear Agriculture and Biotechnology Division, Bhabha Atomic Research Centre, Mumbai, 400085 India; 3 Department of Biotechnology, PES Modern College of Arts Science and Commerce, Ganeshkhind, Pune, 411016 India; 4 Homi Bhabha National Institute, Mumbai, 400094 India

**Keywords:** Agro-morphological parameters, Germplasm diversity, Grain arsenic, Toxicity limit, Quantitative traits

## Abstract

Arsenic (As) is a toxic environmental pollutant and a group-1 carcinogen, posing serious concern for human health. Rice and rice-based food products are considered as one of the major sources for As contamination into the human food-chain. Hence, world-wide concerted research efforts are going on, either to identify and/or develop low-As accumulating rice genotypes. The present study evaluated the natural accessions of rice, comprising of landraces and farmer’s varieties, under the naturally As-contaminated sites of Chhattisgarh, India, which is well-known for its richness in terms of rice production as well as varietal diversity. The wide-range spectrum of grain-As was obtained in 120 selected genotypes, with 90 and 81 accessions having As-accumulation above the WHO permissible limit for white (0.2 mg kg^−1^) and brown (0.3 mg kg^−1^) rice, respectively. The lowest grain-As accumulation among the varieties was seen in cultivated varieties (Badsabhog Sel 1 and Bahadur Sel 1) and landraces (Bastul and Kanaklata), which can be either cultivated or used as suitable donor in breeding programs. Among different agronomic traits, the plant height was identified as most-responsive parameter for assessing As-toxicity. The comparative assessment of agro-morphological traits although showed variations under naturally As-contaminated site; however, no significant correlation was seen in terms of grain-As accumulation, indicating the complexity for on-site field screening of low As-accumulating genotypes. Taken together, the results identified the potential low-As accumulating genotypes and highlighted the grain-As accumulation, as a complex multi-genic trait.

**Supplementary Information:**

The online version contains supplementary material available at 10.1007/s12298-025-01597-z.

## Introduction

Arsenic (As) is a toxic environmental pollutant categorized as Type I carcinogen by International Agency for Research on Cancer (IARC) and is also listed as top-ranked hazardous substance in the United States Agency for Toxic Substance and Disease Registry (ATSDR). One of the recent global surveys have estimated that more than 230 million people worldwide are affected by As-toxicity. This is because groundwater As-levels exceeds beyond 10 µg/L, which is maximum permissible limit as per World Health Organization (WHO 2020). The area under As contamination is continuously increasing due to geogenic as well as anthropogenic sources (Maity et al. 2024; Rokonuzzaman et al. 2022), which is a global concern due to the potential risk associated with human health. The accumulation of As is generally higher in rice than other cereal crops, because of the reductive remobilization of As under anaerobic paddy-field conditions that greatly enhance its bioavailability leading to excessive As accumulation in rice grains (Meharg and Rahman 2003; Neog et al. 2024). Hence, the dietary intake of rice and rice-based food products is considered as a major source of As entry into human food chain (Kumar et al. 2021; Mawari et al. 2022). Due to their geographical location and dietary preferences, Chhattisgarh state of India, has been disproportionately impacted by As-poisoning (Khute et al. 2024; Wakhle et al. 2024). Chhattisgarh is known as the "Rice Bowl of India" because it is one of the country's largest producers of paddy. Besides, it is also known for it's rich heritage, as more than twenty-three thousand rice germplasms with diverse agronomic characters have been documented from this region (Richharia 1981; Sahu et al. 2017). Exploring such natural diversity in rice is crucial for identifying low-As accumulating genotypes because it provides a diverse genetic resource, in terms of As-uptake, translocation, and accumulation. With this background, the present study was conducted to utilize agronomic diversity in rice for the identification of low grain-As accumulating genotypes under the naturally As-contaminated fields of Muleti-tola village, Mohla-Manpur-Ambagarh Chowki District of Chhattisgarh. The results revealed potential low grain-As genotypes and also highlighted that grain-As accumulation is independent of various agro-morphological traits, related to plant growth and crop yield. The findings are important from the broader perspective of cultivating As-safe rice under the field conditions.

## Material and methods

### Details of the experimental sites

The comparative field study was conducted during July–December, 2023 at two independent experimental sites including Indira Gandhi Krishi Vishwavidyalaya (IGKV), Raipur field (control-site; 21.24° N, 81.71° E) and Muleti-tola village of Mohla-Manpur-Ambagarh chowki district (naturally As-contaminated site; 20.78° N, 80.74° E), located in Chhattisgarh, India (Supplementary Fig. 1A). The cultivation area under each experimental site was ~ 0.15 hectare. Refer supplementary table-1 for details regarding the soil properties and meteorological data. At both these experimental sites, soil samples were collected from a total of 50 random locations at 30 cm depth using auger and subjected to the quantification of total-As, pH (Kicińska et al. 2022) and electrical conductivity (EC; Pérez et al. 2014).

### Selection of the germplasm and field testing

On the basis of agronomic traits, a panel of diverse 115 farmer’s varieties/landraces of *indica* rice were collected from the germplasm collection, IGKV, Raipur, Chhattisgarh. In addition, Pooja (Yadav et al. 2021) and MTU1010 (Singhal et al. 2018) were included as a representative for low- and high-As accumulation, respectively, while IR64, Indira Sugandhit Dhan 1 and TN1 were included as yield checks. Seeds of these 120 genotypes were grown at nursery and 21-d old seedlings were independently transplanted at a control- and As-contaminated experimental site, using the augmented randomized complete block design, that fastens the genomic selection with limited seeds by eliminating soil heterogeneity (Zystro et al. 2018). The experimental design consisted of five blocks, each containing a combination of both test entries (new genotypes) and checks (standard varieties). The five checks are repeated in every block, to ensure uniformity and allow comparisons across blocks. However, the test entries were not replicated across blocks, so as to maximize the number of genotypes evaluated, while still maintaining the statistical control through the repeated checks. A total of 20–25 plants/genotype was maintained at both the tested sites (Supplementary Fig. 1B). A similar package-of practice for rice cultivation including irrigation regime, cultivation density, fertigation and tillage practices such as plowing or turning of the soil was maintained across both the experimental sites. A uniform water stagnation of 5 cm was also maintained throughout the growing period to replicate typical flooded paddy-field conditions. At the time of harvest, various agro-morphological parameters like plant height, flag leaf length–width-area, days to 50% flowering, panicle length, number of tillers/effective tillers, biological-grain yield/plant, harvest index, number of filled-unfilled-total grains/plant, spikelet fertility (%), 100 seed weight, seed length:width ratio were recorded manually.

### Quantification of total-As in soil and rice grains

Soil (1 g) and de-husked seed (0.1 g) samples were taken into a clean digestion tube and acid digested using 2 ml of concentrated HNO_3_ (Majumder et al. 2021) and evaporated to dryness at 160 °C using dry heat block. After drying the tubes were allowed to cool at room temperature with final dissolution of the content in 2 ml. The aqueous extract was used for As quantification using Shimadzu AA-6880F atomic absorption spectrophotometer (Shimadzu Corporation, Japan), integrated with a halide vapor generator (HVG-1, Shimadzu Corporation, Japan). The method supports the quantification of total-As in the range of ~ 0.1–100 ppb.

### Statistical analysis

The statistical data analysis was carried out using the “Augmented RCBD” R package (Aravind et al. 2020). Two-way ANOVA and t-test was used to test significant differences between different agro-morphological parameters at *p* ≤ 0.05 and at *p* ≤ 0.01 levels. The error variance was analysed from replicated check genotypes and adjusted from the test genotypes which nullifies the block effect. In two-way ANOVA, the significant values of test vs checks show the interaction between the test genotype group and check genotype group. The adjusted mean obtained from the Augmented RCBD analysis was used as input for the estimation of genetic variability parameters, correlation, regression and principal component analysis (PCA). Various packages like metan, corrplot, FactoMineR and Factoextra in R studio version 4.2.0 was used for creating biplot, scree plot and contribution plots.

## Results and discussion

**Fig. 1:**
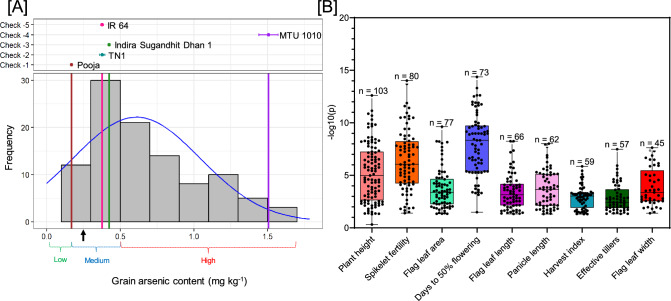
Box plot and grain-As content distribution curve representation of 120 diverse rice genotypes. A set of 120 diverse genotypes were selected for comparative study under control and naturally As-contaminated experimental site. **A** The spectrum in grain-As accumulation was plotted in genotypes along with five check varieties including Pooja, MTU1010, IR 64, Indira Sugandhit Dhan 1 and TN1. The genotypes were classified into three categories as low- (0.02 ± 0.01 mg kg^−1^; n = 15), medium- (0.2 ± 0.1 mg kg^−1^; n = 18) and high- (2 ± 1.5 mg kg^−1^; n = 87) As-accumulating. **B** Comparative assessment of various agro-morphological traits, along with fold-change and p value, under control and naturally As-contaminated site. Replicated values of 5 plants were recorded and significant differences were calculated using t-test. The log10(p) was plotted for different agronomic traits. The values (n) above the box-plot represent the total number of genotypes with significant differences under naturally As-contaminated site, compared with those of control site. Refer Supplementary Table 4, for the raw values

The present field-level study was performed to utilize natural variation in rice germplasm for understanding the correlation between grain As-accumulation and various agronomic traits. Initially, two independent experimental sites were selected including control (total-As below detection limit of < 0.1 ppb)- and As-contaminated (total-As, 14.0 ± 1.2 mg kg^−1^), in the state of Chhattisgarh, India (Supplementary Fig. 1). As per European Union (EU), As-concentration in agriculture soils should not exceed more than 20 mg kg^−1^; however, this generic recommendation may not be appropriate for paddy soil conditions, which are known to have enhanced As-bioavailability to rice (Meharg and Rahman 2003; Neog et al. 2024). In support of this, a meta-analysis has also highlighted that As-concentrations greater than 14 mg kg^−1^ in Asian paddy soil may increase the As-levels, more than the maximum permissible limit in rice grains (Mandal et al. 2021). Considering these facts, the experimental site having 14.0 ± 1.2 mg kg^−1^ total-As was found to be appropriate for the present study. The soil compositional analysis has revealed that realgar (α-As_4_S_4_), pararealgar (AsS or As_4_S_4_), and/or tennantite (Cu_12_As_4_S_13_) are the main minerals that contribute to the As-contamination at the experimental site of Muleti-tola village of Mohla-Manpur-Ambagarh chowki district, Chattisgarh (Das et al. 2013). The soil pH ranged from 6.60–8.22 (neutral-to-slightly alkaline) at the control-site, while, 6.38–7.54 (slightly acidic-to-neutral) at As-contaminated site. Besides, EC at As-contaminated site was found significantly higher (0.38 dS m^−1^) than those of the control site (0.23 dS m^−1^) (Supplementary Table 1A). The slightly acidic pH alongside high EC has been shown to increase As-bioavailability in soil during paddy cultivation (Moreno-Jiménez et al. 2012; Sorlini et al. 2023), further justified the selection of experimental site. In addition, owing to the close proximity, the overall soil properties and meteorological data were similar across the selected experimental sites (Supplementary Table 1B), and hence, the observed differences in agronomic traits could largely be attributed to the As-contamination in the soil. The tested rice germplasm followed a platykurtic distribution curve in terms of grain As-accumulation (Fig. 1A). The platykurtic distribution curve represents a probability distribution with a lower peak and thinner tails compared to a normal distribution. This means the data is more evenly spread out, with fewer extreme values or outliers. Out of 120, a total of 90 and 81 accessions showed As-accumulation above the permissible limit for white (0.2 mg kg^−1^) and brown (0.35 mg kg^−1^) rice, respectively (Åkesson et al. 2012; CODEX 2014). Variation in the level of grain As-accumulation highlighted the genotypic differences between the selected accessions (Supplementary Table 2). Previously, the field trials conducted at As-contaminated fields have also demonstrated genetic variation as a major determining factor for grain-As accumulation in rice (Norton et al. 2012). Several landraces (Bastul and Kanaklata) as well as cultivated varieties (Badsabhog Sel 1 and Bahadur Sel 1) were identified as least As-accumulating rice genotypes. Depending upon the agronomic character and consumer preference, these genotypes can be considered for direct release, or may included as a potential donor in rice breeding program for developing low-As accumulating variety. Apart from grain-As, variation in different agro-morphological traits was also recorded across diverse genotypes (Supplementary Table 2). The mean sum-of-square for these traits was analyzed through ANOVA, at four levels including block-wise, genotype-wise, check genotype and test vs. check genotype. All the tested agro-morphological traits were found to be significant in at least one level, at both the experimental sites, except for the number of filled and unfilled grains, that were found non-significant at the control site (Supplementary Table 3). Further, log_10_ *p* values were computed for agro-morphological traits in individual genotypes showing significant variation in As-contaminated site compared with those of control site (Supplementary Table 4). The traits like plant height (n = 103), spikelet fertility (n = 80) and flag leaf area (n = 77) were identified with significant difference in maximal number of genotypes (Fig. 1B). On the basis of number of genotypes showing significant difference, plant height was identified as the most suitable trait for assessing the As-toxicity in rice. This was further substantiated as the mean plant height showed the maximal level of reduction by 0.15-fold under As-contaminated site, compared with those of control site. However, since plant height is also known to be affected under diverse abiotic stress conditions, hence, it cannot be generalized as specific indicator of As-toxicity. In contrast, other traits such as number of effective tillers, grain yield/plant, number of filled/unfilled spikelet/plant, 100 seed weight and seed length–width ratio were not changed significantly in any of the tested genotypes (Supplementary Table 5).

**Fig. 2:**
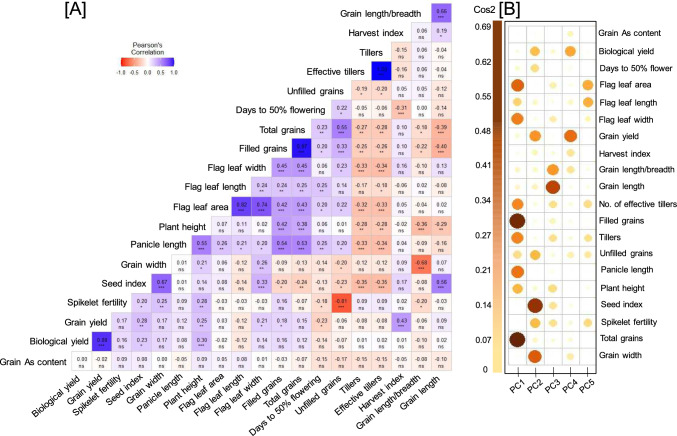
Pearson’s correlation and principal component analysis for understanding inter-dependence between grain-As accumulation and various agro-morphological traits. A total 120 diverse genotypes were independently cultivated under control and naturally As-contaminated site. Various agronomic traits and grain-As accumulation were quantified at the time of harvest. **A** Correlation matrix between recorded agro-morphological traits and grain-As content under naturally-As contaminated site. Single, double and triple asterisk (*) represent *p* value ≤ 0.05, ≤ 0.01 and ≤ 0.001, respectively. While, ns show non-significant. **B** Principal component analysis of recorded parameters using contribution matrix (Cos2) with circles representing contribution of trait to different PC’s. Refer Supplementary Fig. 3, for PCA scree plot, contribution bar plot and biplot under both the experimental sites

To accelerate the breeding program, it is important to understand the inter-relationship between grain-As with quantitative agro-morphological traits, so as to test the feasibility of on-site field screening for low grain-As accumulating rice genotypes. Previously, meta-analysis revealed that aromatic rice grains accumulate lower As, either *indica* or *japonica* (Das et al. 2023). Also, the correlation analysis has been performed in terms of grain-As content with several cooking parameters (Moulick et al. 2022). However, to the best of our knowledge, the comprehensive field analysis of diverse rice genotypes has not been performed to understand dependence of grain-As on various agronomic traits. Towards this objective, Pearson correlation heat matrix was generated for grain As-accumulation and all the predicted variables which provided significant variation within the tested genotypes (Fig. 2A). As expected, significant positive correlation was observed between grain yield/plant and its attributing traits like plant height (0.25**), flag leaf width (0.21*), number of filled spikelets/plant (0.18*), harvest index (0.43***), and 100 seed weight (0.28**). However, no significant correlation was observed for grain-As content with any of the tested traits (Fig. 2A). This was also supported by the regression analysis of grain-As accumulation as dependent trait and various vegetative and reproductive parameters, as independent traits (Supplementary Fig. 2). The present results are in contrast with the earlier study conducted on a total of 18 genotypes, wherein grain morphology was found to be correlated with grain-As accumulation, with lower-As accumulation in fine- than coarse-grain genotypes (Niazi et al. 2022). However, the observed association between grain-As with grain morphology could be due to the selection of inherently low-As accumulating genotypes. In addition, PCA was performed to understand the relative contribution of various traits towards overall data variance. The data reveled that first three PCs contributed 46.5% [PC1 (17.8%), PC2 (16.2%), and PC3 (12.5%)] and 50.1% PC1 (22.3%), PC2 (15.7%), and PC3 (12.1%)] of total variance under control- and naturally As-contaminated site, respectively (Supplementary Fig. 3). PC1 and PC2 contributed more toward yield-attributing traits and could be considered for a rice improvement program, as they are major traits involved in genetic variation. However, grain-As accumulation showed the lowest contribution towards overall data-variance, at the naturally As-contaminated site (Fig. 2B), indicating that grain-As levels did not significantly contribute in segregating the rice genotypes. This further implies that grain-As accumulation is a complex multi-genic trait. In addition, the grain-As accumulation is also known to be under the control of “genotype X environment” interaction (Moulick et al. 2022), which poses another layer of complexity towards the development of As-safe rice.

## Conclusion

Arsenic contamination in rice poses a significant threat to human health, particularly in regions where rice is a staple food. The present study utilized the diverse natural variation in rice to identify low As-accumulating genotypes under the naturally As-contaminated site of Muleti-tola region, Mohala-Manpur-Ambagarh Chowki district of Chhattisgarh, India. A wide-spectrum was seen in terms of grain-As accumulation, indicating the genotypic diversity. The identified low-As accumulating genotypes can be used either for the direct cultivation after reproducible field testing or as a donor in breeding program. The comparative assessment of various agro-morphological traits revealed plant height, as most reliable parameter to assess toxicity under As-contaminated field conditions. Although, grain yield/plant was significantly correlated with yield-determining traits; however, no significant correlation was seen between grain-As content and any of the tested agronomic traits, highlighting the complexity in screening low-As accumulating genotypes. Further, the research findings also advocate the importance of developing novel on-site field screening method(s) for low grain-As accumulating rice genotypes.

## Supplementary Information

Below is the link to the electronic supplementary material.

Supplementary file1 (DOCX 1377 KB)

Supplementary file2 (XLSX 46 KB)

Supplementary file3 (XLSX 99 KB)

Supplementary file4 (DOCX 35 KB)

## References

[CR1] Åkesson MT et al (2012) Proposed draft maximum levels for arsenic in rice (AT STEP 3). Codex Alimentarius Commission. Joint FAO/WHO Food Standards Programme, Codex Committee on Contaminants in Foods, Maastricht, pp 153–316

[CR2] Aravind J et al (2020) Augmented RCBD: analysis of augmented randomised complete block designs. R package version 0.1. 2

[CR3] CODEX AC (2014) Proposed draft maximum levels for arsenic in rice (raw and polished rice). CX. CF 14/8/6. Food and Agriculture Organization of the United Nations, Rome

[CR4] Das S et al (2013) Bioaccessibility and health risk assessment of arsenic in arsenic-enriched soils, Central India. Ecotoxicol Environ Saf 92:252–257 23523002 10.1016/j.ecoenv.2013.02.016

[CR5] Das S et al (2023) Meta-analyses of arsenic accumulation in Indica and Japonica rice grains. Environ Sci Pollut Res 30:58827–58840 10.1007/s11356-023-26729-4 36997784

[CR6] Khute M et al (2024) Arsenic Speciation and Contamination in Cereals from Chhattisgarh, India. J Heavy Met Toxic Dis 9:18816

[CR7] Kicińska A et al (2022) Changes in soil pH and mobility of heavy metals in contaminated soils. Eur J Soil Sci 73:e13203

[CR8] Kumar A et al (2021) Arsenic exposure in Indo Gangetic plains of Bihar causing increased cancer risk. Sci Rep 11:2376 33504854 10.1038/s41598-021-81579-9 PMC7841152

[CR9] Maity S et al (2024) A comprehensive review of arsenic contamination in India with an emphasis on its detection through biosensors and bioremediation from the aqueous system. Environ Qual Manag 33:427–457

[CR28] Majumder Supriya, Banik Pabitra (2021) Inhibition of arsenic transport from soil to rice grain with a sustained field-scale aerobic rice cultural practice. Journal of Environmental Management 279:111620. 10.1016/j.jenvman.2020.111620 33221047 10.1016/j.jenvman.2020.111620

[CR10] Mandal J et al (2021) Meta-analysis enables prediction of the maximum permissible arsenic concentration in Asian paddy soil. Front Environ Sci 9:760125

[CR11] Mawari G et al (2022) Human health risk assessment due to heavy metals in ground and surface water and association of diseases with drinking water sources: a study from Maharashtra, India. Environ Health Insights 16:11786302221146020 36582432 10.1177/11786302221146020 PMC9793032

[CR12] Meharg AA, Rahman MM (2003) Arsenic contamination of Bangladesh paddy field soils: implications for rice contribution to arsenic consumption. Environ Sci Technol 37:229–234 12564892 10.1021/es0259842

[CR13] Moreno-Jiménez E et al (2012) The fate of arsenic in soil-plant systems. Rev Environ Contam Toxicol 1–37 10.1007/978-1-4614-1463-6_1 22057929

[CR14] Moulick D et al (2022) Interrelationship among rice grain arsenic, micronutrients content and grain quality attributes: an investigation from genotype× environment perspective. Front Environ Sci 10:857629

[CR15] Neog N et al (2024) Arsenic contamination in the groundwater of Northeastern India: critical understandings on geotectonic controls and the need for intervention. Curr Opin Environ Sci Health. 38:100539

[CR16] Niazi NK et al (2022) The significance of eighteen rice genotypes on arsenic accumulation, physiological response and potential health risk. Sci Total Environ 832:155004 35381235 10.1016/j.scitotenv.2022.155004

[CR17] Norton GJ et al (2012) Variation in grain arsenic assessed in a diverse panel of rice (Oryza sativa) grown in multiple sites. New Phytol 193:650–664 22142234 10.1111/j.1469-8137.2011.03983.x

[CR18] Pérez I et al (2014) Magnetic susceptibility and electrical conductivity as a proxy for evaluating soil contaminated with arsenic, cadmium and lead in a metallurgical area in the San Luis Potosi State, Mexico. Environ Earth Sci 72:1521–1531

[CR19] Richharia R (1981) An aspect of genetic diversity in rice

[CR20] Rokonuzzaman M et al (2022) Arsenic accumulation in rice: sources, human health impact and probable mitigation approaches. Rice Sci 29:309–327

[CR21] Sahu PK et al (2017) InDel marker based genetic differentiation and genetic diversity in traditional rice (*Oryza sativa* L.) landraces of Chhattisgarh, India. PLoS ONE 12:e0188864 29190790 10.1371/journal.pone.0188864 PMC5708757

[CR22] Singhal VK et al (2018) Accumulation and partitioning of arsenic in rice varieties of Ambagarh Chowki, Rajnandgaon district, Chhattisgarh. IJCS 6:1385–1388

[CR23] Sorlini S et al (2023) Electrochemical treatment of arsenic in drinking water: effect of initial As3+ concentration, pH, and conductivity on the kinetics of oxidation. Clean Technol 5:203–214

[CR24] Wakhle B et al (2024) Multi-element exposure and health risks of grains from Ambagarh Chowki, Chhattisgarh, India. Toxics 13:56 10.3390/toxics13010056 PMC11769171 39853054

[CR25] WHO (2020) Exposure to arsenic: a major public health concern. 2010. WHO Document Production Service, Geneva

[CR26] Yadav P et al (2021) Tracking the time-dependent and tissue-specific processes of arsenic accumulation and stress responses in rice (*Oryza sativa* L.). J Hazard Mater 406:124307 33221079 10.1016/j.jhazmat.2020.124307

[CR27] Zystro J et al (2018) Alternative experimental designs for plant breeding. Plant Breed Rev 42:87–117

