# Supplementary material for: Grain arsenic accumulation is independent of agronomic traits in rice under field conditions
Source: Physiol Mol Biol Plants. 2025 Jun 21;31(10):1793–8. doi: 10.1007/s12298-025-01597-z (PMC12559551; doi:10.1007/s12298-025-01597-z)
Supplement: Supplementary file 1 — Supplementary file1 (DOCX 1377 KB) [file 12298_2025_1597_MOESM1_ESM.docx]

B

A

**Supplementary Figure-1: Geographical locations of the experimental sites and design of the field experiment.** A. The comparative field study was conducted at two independent experimental sites including Indira Gandhi Krishi vishwavidyalaya (I.G.K.V.) field (control-site; 21.24°N, 81.71° E), Raipur and Muleti-tola region of Mohla-Manpur-Ambagarh chowki (naturally As-contaminated site; 20.78°N, 80.74°E). Both the sites were located at Chhattisgarh district of India. B. The field evaluation of a total of 115 genotypes and 5 check varieties was performed using Augmented Randomized Complete Block Design. A total of 20-25 plants/genotype was maintained at both the tested sites. Refer supplementary table-1 for details regarding the soil properties and meteorological data, across both the experimental plots.

**Supplementary Figure 2: Regression analysis of grain-As content with other agro-morphological traits.** Evaluation of coefficient of determination (R2) between grain-As accumulation [dependent trait (y) and other vegetative and reproductive parameters [independent (x)] of 120 diverse genotypes cultivated under As contaminated site. *p*-values computed using R-studio software representing significance.

**Supplementary Figure 3: Scree plot, bar plot and PCA biplot representation of various agro-morphological traits in 120 diverse accessions of rice.** Scree plot representing percentage of explained variance of various agro-morphological traits to different principal components (PCs) under control (A) and naturally As-contaminated contaminated site. Bar plot representation of percentage contribution (Cos2) of various agro-morphological parameters to principal components (PC1 and PC2) cultivated under control (C) and naturally As-contaminated site (D). PCA biplot representation of correlation between recorded parameters and their contribution towards PC1 and PC2 under control (E) and naturally As-contaminated site (F).
