# Supplementary material for: Grain arsenic accumulation is independent of agronomic traits in rice under field conditions
Source: Physiol Mol Biol Plants. 2025 Jun 21;31(10):1793–8. doi: 10.1007/s12298-025-01597-z (PMC12559551; doi:10.1007/s12298-025-01597-z)
Supplement: Supplementary file 4 — Supplementary file4 (DOCX 35 KB) [file 12298_2025_1597_MOESM4_ESM.docx]

**Supplementary Table 1: Details of the soil parameters (A) and meteorological data (B) of control-site (C-Site) at I.G.K.V., Raipur and naturally arsenic-contaminated site (A-Site), Muleti-tola village.**

| **S.No.** | **Soil parameters** | **C-site** | **A-site** |
| --- | --- | --- | --- |
| 1. | Arsenic content (mg kg^-1^) | NTD | 14.0 ± 1.2 |
| 2. | Soil texture | Sandy clay | Clayey |
| 3. | pH | 6.60-8.22 | 6.38-7.54 |
| 4. | EC (dS/m) | 0.23 | 0.38 |

**[A]**

**[B]**

| **Month** | **Average rainfall**  **(mm)** | | **Average relative**  **humidity (%)** | | **Average evaporation**  **(mm)** | | **Average max.**  **temp. (°C)** | | **Average min. temp. (°C)** | | **Average sunshine**  **(hours)** | |
| --- | --- | --- | --- | --- | --- | --- | --- | --- | --- | --- | --- | --- |
|  | **C-site** | **A-site** | **C-site** | **A-site** | **C-site** | **A-site** | **C-site** | **A-site** | **C-site** | **A-site** | **C-site** | **A-site** |
| **June** | 220.4 | 225.4 | 52 | 54 | 240.1 | 242.1 | 39.5 | 38.2 | 27.0 | 25.0 | 6.5 | 6.3 |
| **July** | 338.8 | 342.7 | 80 | 79 | 108.6 | 104.6 | 32.2 | 32.4 | 25.7 | 24.8 | 3.9 | 3.5 |
| **August** | 150.6 | 151.4 | 79 | 81 | 108.3 | 111.3 | 31.7 | 31.9 | 25.1 | 23.7 | 4.1 | 4.2 |
| **September** | 70.6 | 69.2 | 82 | 81 | 85.9 | 83.9 | 31.4 | 32.3 | 25.0 | 26.0 | 3.3 | 3.1 |
| **October** | 9.4 | 8.9 | 66 | 64 | 115.9 | 117.9 | 32.5 | 32.6 | 20.9 | 20.9 | 7.9 | 7.6 |
| **November** | 0 | 0 | 59.5 | 61 | 89 | 85 | 29.9 | 27.5 | 13.8 | 12.9 | 7.5 | 7.8 |
| **December** | 17.8 | 16.5 | 66 | 65 | 75.4 | 77.4 | 27.3 | 25.6 | 13.8 | 12.4 | 5.3 | 5.4 |

Supplementary Table 3: ANOVA table representing mean sum-of-square to highlight significant/non-significant difference in treatments, checks and blocks for agro-morphological traits of 120 diverse genotypes (115 genotypes + 5 checks) under control and As-contaminated site. Significant variation signifies trait selection suitability for further studies. Single, double and triple asterisk (*) represent p-value ≤ 0.05, ≤ 0.01 and ≤ 0.001, ns is non-significant for respective traits. [Df – Degree of freedom, DTF - Days to 50% flowering, PH - Plant height, FLL - Flag leaf length, FLW - Flag leaf width, FLA - Flag leaf area, PaL - Panicle length, NoT - Number of tiller, NoET - Number of effective tiller, BY - Biological yield, GY - Grain yield, HI - Harvest index, NoFG - Number of filled grains, NoUG - Number of unfilled grains, TG - Total grains, SPF - Spikelet fertility, LG - Grain length, WG - Grain width, L/B -grain Length/Breadth, SI - Seed index, AsG - Grain Arsenic]

| **ANOVA table (control condition)** | | | | | | | | | | | | | | | | | | | | | |
| --- | --- | --- | --- | --- | --- | --- | --- | --- | --- | --- | --- | --- | --- | --- | --- | --- | --- | --- | --- | --- | --- |
|  | **Df** | **DTF** | **PH** | **FLL** | **FLW** | **FLA** | **PaL** | **NoT** | **NoET** | **BY** | **GY** | **HI** | **NoFG** | **NoUG** | **TG** | **SPF** | **LG** | **WG** | **L/B** | **SI** | **AsG** |
| **Block**  (Eliminating genotypes) | 4 | 307.61 ** | 2562.34 ** | 47.51 ** | 0.09 ⁿˢ | 85.80 * | 28.17 ** | 30.17 ** | 34.79 ** | 13350.41 ** | 3434.27 ** | 0.03 ** | 5013.00 ⁿˢ | 255.85 ⁿˢ | 5873.41 ** | 53.23 ⁿˢ | 1.28 ** | 0.17 ** | 0.61 ** | 0.20 ** |  |
| **Genotypes**  (Eliminating blocks) | 119 | 199.29 ** | 1191.47 ** | 52.77 ** | 0.09 * | 189.19 ** | 13.54 ** | 13.61 ** | 12.65 ** | 9132.03 ** | 3434.76 ** | 0.01 * | 2817.44 ⁿˢ | 637.81 ⁿˢ | 3463.70 ** | 135.30 ⁿˢ | 1.34 ** | 0.14 ** | 0.42 ** | 0.38 ** |  |
| **Genotypes: Check** | 4 | 217.29 ** | 670.28 ** | 68.73 ** | 0.45 ** | 613.97 ** | 8.17 ** | 33.98 ** | 34.15 ** | 46279.43 ** | 17199.84 ** | 0.04 ** | 4920.96 ⁿˢ | 2578.08 ** | 11558.51 ** | 703.75 ⁿˢ | 2.43 ** | 0.12 ** | 0.25 * | 0.18 ** |  |
| **Genotypes:**  **Test vs. Check** | 115 | 198.66 ** | 1209.60 ** | 52.21 ** | 0.08 * | 174.42 ** | 13.73 ** | 12.91 ** | 11.90 ** | 7839.95 ** | 2955.97 ** | 0.01 ⁿˢ | 2744.27 ⁿˢ | 570.32 ⁿˢ | 3182.14 ** | 115.53 ⁿˢ | 1.30 ** | 0.14 ** | 0.43 ** | 0.38 ** |  |
| **Residuals** | 16 | 1.46 | 12.31 | 1.92 | 0.04 | 20.88 | 0.53 | 1.15 | 0.86 | 1366.53 | 651.49 | 0.00 | 2463.77 | 539.34 | 702.04 | 254.32 | 0.09 | 0.02 | 0.05 | 0.00 |  |
| **ANOVA table (As contaminated site)** | | | | | | | | | | | | | | | | | | | | | |
| **Block**  (Eliminating genotypes) | 4 | 370.67 ** | 817.00 ** | 20.42 ⁿˢ | 0.18 * | 213.98 ⁿˢ | 15.00 ** | 104.83 ** | 106.78 ** | 12867.01 ** | 3129.67 ** | 0.02 * | 6058.58 ** | 531.17 * | 9620.92 ** | 79.74 ⁿˢ | 1.25 ** | 0.19 ** | 0.64 ** | 0.19 * | 0.19 ** |
| **Genotypes**  (Eliminating blocks) | 119 | 166.44 ** | 722.36 ** | 40.74 * | 0.06 ⁿˢ | 208.15 ⁿˢ | 7.82 ** | 13.03 ⁿˢ | 12.83 ⁿˢ | 9261.32 ** | 3472.18 ** | 0.01 ⁿˢ | 2569.98 * | 229.44 ⁿˢ | 3347.55 * | 51.17 ⁿˢ | 1.36 ** | 0.14 ** | 0.43 ** | 0.37 ** | 0.22 ** |
| **Genotypes: Check** | 4 | 790.46 ** | 142.91 ⁿˢ | 14.52 ⁿˢ | 0.04 ⁿˢ | 216.59 ⁿˢ | 4.55 ⁿˢ | 15.64 ⁿˢ | 13.81 ⁿˢ | 50897.00 ** | 17741.66 ** | 0.01 ⁿˢ | 606.45 ⁿˢ | 347.77 ⁿˢ | 1109.94 ⁿˢ | 120.34 ⁿˢ | 2.72 ** | 0.08 * | 0.50 ** | 0.14 * | 1.41 ** |
| **Genotypes:**  **Test vs. Check** | 115 | 144.73 ** | 742.52 ** | 41.65 * | 0.06 ⁿˢ | 207.85 ⁿˢ | 7.94 ** | 12.94 ⁿˢ | 12.79 ⁿˢ | 7813.13 ** | 2975.85 ** | 0.01 ⁿˢ | 2638.28 * | 225.32 ⁿˢ | 3425.38 * | 48.76 ⁿˢ | 1.31 ** | 0.14 ** | 0.43 ** | 0.38 ** | 0.17 ** |
| **Residuals** | 16 | 6.43 | 65.23 | 18.00 | 0.06 | 165.36 | 1.64 | 12.89 | 12.53 | 1098.40 | 582.96 | 0.01 | 1090.82 | 152.26 | 1555.78 | 44.61 | 0.12 | 0.02 | 0.05 | 0.04 | 0.00 |
| **ⁿˢ P > 0.05; * P <= 0.05; ** P <= 0.01** | | | | | | | | | | | | | | | | | | | | | |

Supplementary Table 5: Overall mean table for comparison of agro-morphological traits evaluated under control and naturally As-contaminated site. A diverse 120 genotypes comprising landraces and popular cultivars of Chhattisgarh and West-Bengal were selected for comparative agro-morphological evaluation under control and naturally As-contaminated site. Germinated seeds were grown at controlled condition and 21 days old seedlings were transplanted under control and naturally As-contaminated site and cultivated till maturity using common agricultural practices. All the quantitative traits were evaluated including harvesting of seeds for grain As-quantification. Table represents the comparative analysis of overall mean with standard deviation of all the recorded parameters.

| **S. No.** | **Agro-morphological Traits** | **Overall mean**  **(Control site)** | **Overall mean**  **(As-contaminated site)** |
| --- | --- | --- | --- |
| 1. | Days to 50% Flowering | 94.43 ± 14.11 | 86.73 ± 12.70 |
| 2. | Plant height (cm) | 128.88 ± 32.97 | 109.546 ± 25.03 |
| 3. | Flag leaf length (cm) | 34.83 ± 7.22 | 31.8048 ± 6.48 |
| 4. | Flag leaf width (cm) | 1.53 ± 0.27 | 1.58 ± 0.26 |
| 5. | Flag leaf area | 42.28 ± 13.21 | 40.3 ± 14.79 |
| 6. | Panicle length (cm) | 25.78 ± 3.72 | 24.10 ± 2.95 |
| 7. | Number of tillers/plants | 12.89 ± 3.67 | 12.257 ± 4.95 |
| 8. | Number of effective tillers/plants | 11.98 ± 3.50 | 11.97 ± 4.92 |
| 9. | Biological yield (g) | 278.30 ± 92.04 | 270.54 ± 93.69 |
| 10. | Grain yield/plant (g) | 174.12 ± 56.28 | 159.30 ± 57.51 |
| 11. | Harvest index | 0.63 ± 0.09 | 0.59 ± 0.10 |
| 12. | Number of filled spikelet’s/plant | 148.87 ± 56.50 | 139.91 ± 52.84 |
| 13. | Number of unfilled spikelet’s/plants | 20.45 ± 25.14 | 19.24 ± 15.53 |
| 14. | Total number of spikelet’s/ plants | 168.38 ± 58.41 | 159.14 ± 59.83 |
| 15. | Spikelet fertility (%) | 89.05 ± 12.71 | 88.22 ± 7.50 |
| 16. | Grain length (mm) | 8.27 ± 1.12 | 8.28 ± 1.13 |
| 17. | Grain width (mm) | 2.60 ± 0.38 | 2.60 ± 0.38 |
| 18. | Grain length/width (ratio) | 3.24 ± 0.66 | 3.24 ± 0.65 |
| 19. | 100 seed weight (g) | 2.15 ± 0.61 | 2.18 ± 0.63 |
| 20. | Grain As content (mg kg^-1^) | 0 | 0.61 ± 0.43 |
